# The needle electromyography findings in the neurophysiological classification of ulnar neuropathy at the elbow

**DOI:** 10.3906/sag-1910-59

**Published:** 2020-06-23

**Authors:** Halit FİDANCI, İlker ÖZTÜRK, Ahmet Candan KÖYLÜOĞLU, Mehmet YILDIZ, Şencan BUTURAK, Zülfikar ARLIER

**Affiliations:** 1 Department of Clinical Neurophysiology, Adana City Training & Research Hospital, Adana Turkey; 2 Department of Neurology, Adana City Training & Research Hospital Turkey

**Keywords:** Needle electromyography, neurophysiological classification, ulnar neuropathy at the elbow

## Abstract

**Background/aim:**

Although ulnar neuropathy at the elbow (UNE) is the second most common entrapment mononeuropathy, there are few reports on its neurophysiological classification. In this study, we tried to find out the role of needle electromyography (EMG) in the neurophysiological classification of UNE.

**Materials and methods:**

UNE patients who met the clinical and neurophysiological diagnostic criteria and healthy individuals were included in this study. Reference values of nerve conduction studies were obtained from healthy individuals. Needle EMG was performed to all UNE patients. According to the neurophysiological classification proposed by Padua, UNE patients were classified as mild, moderate, and severe.

**Results:**

Thirty-one controls and thirty-five UNE patients were included in the study. There was mild UNE in 23 patients, moderate UNE in 8, and severe UNE in 4. Abnormal needle EMG findings were present in all patients with moderate and severe UNE and in 12 patients with mild UNE.

**Conclusion:**

Abnormal needle EMG findings are seen in most of the UNE patients. Therefore, it is not practical to use needle EMG findings in the neurophysiological classification. Needle EMG abnormalities may also be present in patients with mild UNE due to axonal degeneration or motor conduction block.

## 1. Introduction

Ulnar neuropathy at the elbow (UNE) is a common mononeuropathy, and nerve conduction study is important for its diagnosis. The American Association of Neuromuscular and Electrodiagnostic Medicine recommends using the following criteria in the diagnosis of UNE: slowing of motor nerve conduction velocity (NCV) at the elbow segment, increased velocity difference between the motor NCV of the forearm and elbow segments (FEVD), a 20% reduction in the compound muscle action potential (CMAP) amplitude or a CMAP morphology change above the elbow compared to the CMAP obtained by stimulating the ulnar nerve below the elbow [1,2]. Short-segment motor nerve conduction studies across the elbow are recommended and are considered as gold standard for diagnosis of UNE and localization of the lesion [3–6].

 Although UNE is the second most common entrapment mononeuropathy following carpal tunnel syndrome and there are many classifications and questionnaires according to clinical features or examination findings [7–9], there are few publications on its neurophysiological classification [10,11]. The UNE classification suggested by Padua et al. seems to be a good one, but it does not include needle electromyography (EMG) findings [10]. In another recently proposed classification, needle EMG findings were included in addition to nerve conduction studies, and this article describes the classification based on two cases [11]. We planned our study based on the classification proposed by Padua et al. and aimed to determine the role of needle EMG in the neurophysiological classification of UNE. 

## 2. Materials and methods

### 2.1. Study design and subjects

Healthy individuals and UNE patients older than 18 years of age who applied to the Neurology Department of Adana City Training & Research Hospital (ACTRH) between November 2018 and June 2019 were included in the study. Written informed consent was obtained from all participants. The study was approved by the Ethics Committee of ACTRH (number: 25/335). Median, ulnar, tibial, peroneal, superficial peroneal, and sural nerve conduction studies were performed on all subjects by a clinical neurophysiologist and a neurologist. Nerve conduction studies were performed bilaterally on the upper extremities of all UNE patients. First, we formed a control group consisting of healthy individuals for the upper and lower limits of normal values for nerve conduction studies. If the participant had any of the following, they were not included in the control group: paresthesia on the extremities, muscle weakness, elbow pain, history of elbow fracture or elbow surgery, a neurodegenerative disease, mononeuropathies, a disorder that could cause polyneuropathy such as diabetes mellitus, a neuromuscular disorder, or abnormality in neurological examination such as decreased tendon reflexes. In addition, individuals with a family history of neurodegenerative disease or hereditary polyneuropathy were excluded from the study. The UNE group consisted of patients who met both clinical and neurophysiological criteria [12,13]. The clinical criteria were two or more of the following: 1) subjective paresthesia or numbness of the fourth and fifth fingers, 2) abnormalities in the sensory area of ​​the ulnar nerve detected on neurological examination, 3) weakness of the ulnar nerve innervated muscles detected on neurological examination. To meet the neurophysiological criteria, the latency difference or CMAP amplitude drop obtained from the short-segment motor nerve conduction study had to be higher than the upper limits of normal values, or ulnar motor NCV at the elbow segment had to be slower than the lower limits of normal value. The UNE patients had the same exclusion criteria as the controls, except for clinical and neurological examination findings compatible with ulnar neuropathy. Individuals suggestive of Martin-Gruber anastomosis in nerve conduction studies were excluded. In case of abnormalities in ulnar nerve innervated muscles, other muscles such as abductor pollicis brevis were also examined for differential diagnosis. Clinical and needle EMG findings compatible with cervical radiculopathy or brachial plexopathy were excluded from the study. The Turkish version of disabilities of the arm, shoulder, and hand (DASH) questionnaire was used to measure upper extremity disability and symptoms [14]. The DASH questionnaire consisting of 30 questions was administered to all patients, and the disability/symptom score was calculated.

### 2.2. Electrodiagnostic tests

All studies were performed with Cadwell Sierra Summit EMG unit (Cadwell laboratories, Kennewick, Washington, USA). Surface electrodes were used for stimulation and recording. Nerve conduction studies were performed when the extremities were above 32 °C. Cold extremities were heated. Band-pass filters for sensory and motor nerve conduction studies were set at 20 Hz to 2 kHz and 20 Hz to 10 kHz, respectively. Nerves were stimulated supramaximally. Sensitivity was 2 mV/division, and sweep speed was 5 ms/division in motor conduction studies. In sensory nerve conduction studies, sensitivity and sweep speed were set to 10 µV and 1 ms/division, respectively. CMAP and sensory nerve action potential (SNAP) amplitudes were measured from peak to peak. Sensory NCV was calculated using peak latency, except that superficial peroneal nerve velocity was calculated using onset latency. Median sensory nerve conduction studies were performed orthodromically by stimulating the 1st, 2nd, 3rd fingers, and the palm. Superficial peroneal and sural nerve conduction studies were performed antidromically. Motor nerve stimulation was performed 5, 8, and 10 cm proximal to the active recording electrode at the wrist and ankle to obtain the median, peroneal, and tibial nerve CMAP, respectively. Minimal F-wave latencies were determined by evaluating at least 10 responses. The ulnar motor nerve conduction study was performed by recording from abductor digiti minimi (ADM) and first dorsal interosseous (FDI) muscles. Buschbacher’s method was used for ulnar motor nerve conduction [15,16]. Nerve conduction studies were performed with the arm at 45° abduction and the elbow at 90° flexion. Distal stimulation point was 5 cm proximal to active electrode on ADM muscle to obtain ulnar CMAP and 12 cm (pathway of ulnar nerve was measured) proximal to active electrode on FDI muscle. Proximal stimulation points were 4 cm distal and 6 cm proximal to the medial epicondyle. Short-segment motor nerve conduction was performed based on Kanakamedala’s method [4]. A line was drawn between the medial epicondyle and olecranon (E), and points were placed at both distal (D2, D4) and proximal (P2, P4, P6) at 2 cm intervals from E. Stimulation was performed on these six points. Ulnar sensory nerve conduction study was performed orthodromically by stimulation of the 5th finger. Forearm and upper arm mixed nerve conduction studies were also performed. Based on the classification used by Padua et al., the neurophysiological classification of UNE was made as follows [10]: 1) Negative UNE: normal ulnar nerve conduction study, 2) Mild UNE: slowing of ulnar motor NCV across the elbow, 3) Moderate UNE: slowing of ulnar motor NCV across the elbow and reduction of the SNAP amplitude, 4) Severe UNE: slowing of ulnar motor NCV across the elbow and absence of SNAP (5th finger-wrist segment), 5) Extreme UNE: absence of ulnar CMAP and SNAP (5th finger-wrist segment). Extreme UNE patients were excluded because localization could not be determined. In addition, negative UNE patients were not included in the UNE group. Needle EMG was performed visually using concentric EMG needle electrode (length = 50mm, diameter = 0.46 mm, Bionen medical devices, Florence, Italy). Concentric needle EMG of ADM, FDI, flexor digitorum profundus of fourth-fifth fingers (FDP) and flexor carpi ulnaris (FCU) muscles were performed in all UNE patients. High-pass and low-pass filters were set at 10 Hz and 10 kHz, respectively. Sensitivity was 50µV/division for the analyses of spontaneous activity and 200–1000 µV/division for motor unit action potential (MUP) evaluation. Sweep speed was 10 ms/division for the analyses of spontaneous activity and MUPs. Positive sharp waves (PSW) and fibrillations were carefully evaluated. At least 10–20 MUPs (according to patients’ tolerability) were recorded during mild muscle contraction. MUP was considered chronic neurogenic if: MUP peak to peak amplitude was ≥4 mV and/or MUP duration was ≥15 ms. 

### 2.3. Statistical analysis

The Shapiro–Wilk test was used to determine the distribution of the data. Group comparisons were made using the Mann–Whitney U test for independent samples. Pearson’s chi-squared test was used to analyze categorical variables. Spearman’s test was used for correlation analysis. Mean ± standard deviation (SD) and median of numeric data were calculated for descriptive statistics. Upper and lower limits were calculated as mean ± 2 SD for normally distributed variables and as 2.25th or 97.75th percentile values for data that were not normally distributed [17]. A P-value of less than 0.05 was considered significant. SPSS (IBM Corp; Armonk, NY, USA) 22.0 was used to perform the statistical analysis.

## 3. Results

The control and UNE groups consisted of 31 and 35 individuals, respectively. Thirteen of the controls (42%) and twelve of the UNE patients were female (34%). The mean ages of the control and UNE groups were 37.8 ± 11.7 (range: 18–64) and 41.6 ± 15.2 (range: 18–77), respectively. The mean height, weight, and body mass index (BMI) of the controls were 170.3 ± 8.1 cm, 74.8 ± 11.6 kg, and 25.8 ± 3.8 kg/m2, respectively, and these values ​​were 172.1 ± 8.9 cm, 76.3 ± 13.1 kg, 25.7 ± 3.8 kg/m2 for the UNE group, respectively. No statistically significant difference was found between the groups in terms of age, sex, height, weight, and BMI. Nerve conduction study was performed on the right upper and lower extremities in 14 of the controls (45%). Twelve of UNE patients (34%) had ulnar neuropathy on the right side. The mean duration of symptoms was 7.6 ± 12.1 (range: 1–60) months. All UNE patients had paresthesia at the 4th and 5th fingers. Twenty-one (60%) patients had paresthesia on the palm. Fourteen patients (40%) had elbow or forearm pain. In neurological examination, sensory abnormalities in the 4th/5th fingers and ulnar side of palm were found in 33 (94%) and 25 patients (71%), respectively. Neurological examination revealed weakness in ADM and FDI in 13 (37%) and 14 (40%) patients, respectively. Atrophy was found in ADM muscle in 4 (11%) patients and in FDI muscle in 6 (17%) patients. Elbow flexion compression test and Tinel’s test were positive in 21 (60%) and 13 (37%) patients, respectively.

The upper or lower limits of reference values for nerve conduction studies obtained from the controls were as follows; ulnar SNAP 5th finger–wrist segment amplitude 7.1 µV, NCV 38.8 m/s; ulnar mixed nerve forearm, upper arm segments amplitude 7.0 µV, 4.4 µV, NCV 47.9 m/s, 49.6 m/s; ulnar nerve distal CMAP latency 2.9 ms (ADM), 4.9 ms (FDI), amplitude 8.0 mV (ADM), 6.4 mV (FDI); ulnar motor NCV wrist–below elbow segment 52 m/s (ADM), 50.9 m/s (FDI); ulnar motor NCV below elbow–above elbow segment 43 m/s (ADM), 45.7 m/s (FDI). The upper reference limits of the latency difference obtained from ADM/FDI muscles across D4-D2, D2-E, E-P2, P2-P4, P4-P6 segments were 0.6/0.5, 0.6/0.7, 0.7/0.8, 0.5/0.7, 0.6/0.5 ms, respectively. In the short segment ulnar motor nerve conduction study recorded from ADM and FDI muscles, the upper limits of normal values obtained from controls for amplitude reduction at D2, E, P2, P4, P6 points were 10%, 10%, 10%, 13%, 14% (ADM) and 6%, 22%, 22%, 26%, 27% (FDI), respectively. The upper reference limit of amplitude drop in percentage across elbow segment was 14.9% for ADM muscle and it was 22.7% for FDI muscle. The upper reference limits of FEVD recorded from ADM and FDI muscles were 15.0 and 14.3 m/s, respectively. Considering the reference values obtained from healthy individuals, 2 patients had carpal tunnel syndrome in addition to ulnar neuropathy, and 1 patient had sural and median nerve SNAP abnormalities in addition to ulnar neuropathy, and these three patients were excluded from the UNE group. Although nine patients had clinical findings, these patients were excluded due to normal nerve conduction studies. Nerve conduction study abnormalities in UNE ​patients ​are shown in Table 1. The PSW and/or fibrillation potentials in ADM, FDI, FDP, FCU muscles were observed in 10, 15, 2, and 3 patients, respectively. Neurogenic changes in ADM, FDI, FDP, FCU muscles were observed in 14, 12, 7, and 10 patients, respectively. According to the classification of Padua et al., 23 patients had mild UNE, 8 patients had moderate UNE, and 4 patients had severe UNE. All patients in the moderate and severe UNE groups had needle EMG abnormalities in at least one of the ADM, FDI, FDP, and FCU muscles. Needle EMG of these four muscles was normal in 11 patients in the mild UNE group. Needle EMG findings in each UNE group are shown in Table 2. Table 3 shows the comparison of nerve conduction studies in mild UNE patients with normal and abnormal needle EMG findings. Latency difference was prolonged in the E-P2 (29 patients), D2-E (5 patients), and P2-P4 (1 patient) segments in UNE. Four of five patients with prolonged latency in the D2-E segment were in the severe UNE group, and one was in the mild UNE group. Needle EMG findings of these five patients were abnormal in at least one muscle. The mean ± SD (median) values of DASH scores were 23.3 ± 17.1 (21), 44 ± 18 (50), and 43 ± 21 (40) in the mild, moderate, and severe UNE groups, respectively. There was a positive correlation between DASH scores and neurophysiological classification of UNE (P = 0.003, r = 0.506).

**Table 1:** Abnormal nerve conduction study in UNE patients.

	Number of extremities with abnormal values (%)
Sensory nerve conduction	
5th digit-wrist segment SNAP amplitude <7.1 µV or velocity <38.8 m/s	12 (34)
Mixed nerve conduction	
Forearm segment amplitude < 7.0µV or velocity < 47.9m/s	14 (40)
Upper arm segment amplitude < 4.4µV or velocity < 49.6m/s	16 (46)
Motor nerve conduction CMAP amplitude –ADM < 8.0 mV/ FDI < 6.4mV	6 (17) / 5 (14)
Motor nerve conduction (Elbow segment)	
Velocity (ADM) < 43 m/s / (FDI) < 45.7 m/s	15 (43) / 20(57)
FEVD (ADM) > 15 m/s / (FDI) > 14.3 m/s	17(49) / 20(57)
Amplitude drop (ADM) > 14.9% / (FDI) > 22.7%	13(37) / 12(34.0)
Short-segment ulnar motor nerve conduction	
Abnormal latency difference (ADM) / (FDI)	31 (89) / 30 (86)
Abnormal amplitude drop (ADM) / (FDI)	14 (40) / 12(34)

SNAP: sensory nerve action potential, FEVD: the velocity difference between the motor NCV of the forearm and elbow segments, ADM: abductor digiti minimi, FDI: first dorsal interosseous.

**Table 2:** Needle EMG findings in each UNE group.

Needle EMG abnormality (fibrillation potentials/PSW or neuropathic changes)	Mild UNE patients n = 23	Moderate UNE patients n = 8	Severe UNE patients n = 4	All UNE patients (%) n = 35
ADM	10	5	4	19 (54%)
FDI	10	7	4	21 (60%)
FDP	4	2	2	8 (23%)
FCU	5	2	4	11(31%)
ADM or FDI or FDP or FCU (%)	12 (52%)	8 (100%)	4 (100%)	24 (69%)

EMG: electromyography, ADM: abductor digiti minimi, FDI: first dorsal interosseous, FDP: flexor digitorum profundus of fourth-fifth fingers, FCU: flexor carpi ulnaris, PSW: positive sharp waves.

**Table 3:** Comparison of ulnar nerve conduction studies in mild UNE patients with normal and abnormal needle EMG findings.

	Abnormal needle EMG n = 12 mean ± SD (median)	Normal needle EMG n = 11 mean ± SD (median)	P-value
5th digit-wrist segment SNAP amplitude (µV)	10.2 ± 2.3 (9.9)	11.4 ± 4.0(9.6)	0.689
5th digit-wrist segment SNAP velocity (m/s)	45.1 ± 5.2 (45.0)	43.9 ± 4.2 (44.0)	0.599
Forearm segment mixed nerve amplitude (µV)	10.6 ± 5.9 (9.8)	32.0 ± 22.7 (21.1)	0.004
Forearm segment mixed nerve velocity (m/s)	53.4 ± 6.3 (52.2)	53.5 ± 4.4 (53.0)	0.751
Upper arm segment mixed nerve amplitude (µV)	7.8 ± 9.7 (5.9)	11.9 ± 7.5(10.9)	0.182
Upper arm segment mixed nerve velocity (m/s)	56.2 ± 1.8 (56.0)	55.3 ± 5.3 (55.8)	0.571
CMAP amplitude-ADM (mV)	11.4 ± 2.6 (11.5)	13.6 ± 2.9 (13.2)	0.148
Elbow segment Velocity-ADM (m/s)	40.1 ± 6.8 (41.0)	52.2 ± 6.6 (53.0)	0.001
FEVD-ADM (m/s)	20.2 ± 10.1 (17.5)	10.6 ± 5.6 (12.0)	0.016
Amplitude drop-ADM (below elbow - above elbow) (%)	52.4 ± 39.3 (59.2)	5.8 ± 10.6 (3.4)	0.005
F-wave latency-ADM (ms)	29.3 ± 2.4 (28.8)	26.5 ± 2.2 (26.5)	0.016
CMAP amplitude-FDI (mV)	13.6 ± 4.9 (12.4)	17.3 ± 5.9 (17.1)	0.140
Elbow Velocity-FDI (m/s)	39.0 ± 6.7 (38.0)	47.6 ± 8.6 (45.0)	0.015
FEVD-FDI (m/s)	20.2 ± 6.4 (21.0)	14.7 ± 8.9 (15.0)	0.052
Amplitude drop-FDI (below elbow – above elbow) (%)	61.2 ± 37.2 (82.0)	8.4 ± 10.3 (5.0)	0.001

n: number of extremities, SD: standard deviation, CMAP: compound muscle action potential, SNAP: sensory nerve action potential, FEVD: The velocity difference between the motor NCV of the forearm and elbow segments, ADM: abductor digiti minimi, FDI: first dorsal interosseous. A P-value of less than 0.05 was considered significant (given in bold).

## 4. Discussion

We determined the upper or lower limits of median, ulnar, tibial, peroneal, and sural nerves from healthy individuals before including UNE patients in the study. Thus, two UNE patients with carpal tunnel syndrome and one with abnormalities in sural and median nerve conduction studies were not included in the UNE group. Needle EMG findings in these three patients could be confusing. Nine patients (21%) had clinical findings suggestive of UNE, but unfortunately, these patients had normal nerve conduction studies. This finding was similar to other studies [10,12]. To increase sensitivity in the diagnosis of UNE, it is recommended by some to perform ultrasonography or ulnar motor nerve conduction study recorded from the FDI muscle [6,12,18]. We also performed an ulnar motor nerve conduction study by recording from both ADM and FDI muscles. In the short-segment ulnar motor nerve conduction study recorded from ADM muscle, abnormal latency difference or amplitude drop was not detected in 4 of 35 patients. The diagnosis of UNE in these four patients was detected in short-segment motor nerve conduction study recorded from FDI muscle. Therefore, we recommend performing ulnar motor nerve conduction study from both ADM and FDI muscles in patients with clinical suspicion. Although there was no significant difference in the sensitivity of motor nerve conduction studies recorded from ADM and FDI muscles in previous studies, nerve conduction studies recorded from two muscles rather than from one muscle would lead to more patients diagnosed with UNE [6,12].

Similar to previous studies, paresthesia was the most common symptom in the fourth and fifth fingers, and the most common abnormality in neurological examination was sensory abnormalities in these fingers [6,10,12,19]. Elbow or forearm pain was less frequent than fourth and fifth finger paresthesia. The DASH questionnaire appears to be a questionnaire that can assess UNE symptoms well. A positive correlation between the DASH scores and the neurophysiological classification of UNE was also found in previous studies such as this study, ant this finding was important in showing that the DASH questionnaire can be used in UNE [10]. Short-segment ulnar motor nerve conduction study at the elbow is considered the gold standard for the diagnosis of UNE [3-6]. For this reason, we planned to include patients with abnormalities in the short-segment (5 × 2 cm) ulnar motor nerve conduction study. The reference values we found for latency differences and amplitude drop in 2 cm segments were similar to those of previous studies [4,13,19]. In controls, the most prominent latency changes at the elbow were observed in D2-E and E-P2 segments, similar to the findings of other studies [3–5,13,19]. In our study, ulnar nerve entrapment in the E-P2 segment was found in 29 of 35 UNE patients. Retroepicondylar entrapment of the ulnar nerve is more common than other sites in UNE [6,18]. In previous studies, a lesion in humeroulnar aponeurotic arcade (HUA) in UNE is seen in 15–20% of patients [3,6,18,20]. The entrapment site was HUA in 5 patients (14%) in our study. Four of these five patients were in the severe UNE group and all of these five patients had abnormal EMG findings in at least one muscle. There are studies showing that axon damage is higher in entrapment in HUA and that demyelination is more prominent in entrapment in retroepicondylar groove, and these studies support our findings [6,18]. UNE can also be classified by lesion site, axonal damage is more pronounced in HUA. 

In UNE, it is known that hand muscles innervated by ulnar nerve are more affected than proximal muscles [6,12,21]. Similarly, in our study, ADM and FDI muscles were more affected than FDP and FCU muscles. This pattern of involvement can be explained by the topographic distribution of the ulnar nerve fascicles [22]. Forearm muscles may be better protected than hand muscles. In a study by Eliaspour et al., muscle involvement was much higher than ours in UNE. In the study of Eliaspor, the percentage of involvement of ADM, FDI, FCU, and FDP muscles in UNE patients were 91.3%, 91.9%, 64.9%, and 56.8%, respectively [21]. This difference can be explained by methodological reasons and patient characteristics. In this study, the ulnar nerve CMAP or SNAP abnormalities were much higher than in our study, and in contrast to our study, needle EMG abnormality was one of the UNE diagnostic criteria. In addition, we did not include the presence of motor unit recruitment abnormality in neurogenic MUP criteria. For these reasons, muscle involvement rates were lower in our study. The percentage of muscle involvement was similar to that of the study by Beekman [12]. The symptom duration of the patients in our study ranged from 1 to 60 months. Therefore, in the case of one of the active denervation or neurogenic changes, needle EMG would be appropriate to be considered abnormal. All patients with moderate and severe UNE had at least one muscle abnormality innervated by ulnar nerve. In mild UNE, needle EMG was normal in 11 of 23 patients. In mild UNE patients with abnormal EMG findings, ulnar nerve motor NCV across the elbow was significantly slower, amplitude drop at the elbow and FEVD and ulnar F-wave latency was significantly higher than in mild UNE patients with normal needle EMG findings. These findings may indicate that needle EMG abnormalities may be due not only to axonal damage but also to the motor conduction block. This may be due to loss of a small amount of motor axons in the region where there is severe demyelination [23]. In addition, amplitude of forearm ulnar mixed nerve action potential was significantly lower in mild UNE patients with abnormal EMG compared to those with normal EMG. All these findings may suggest that electrodiagnostic tests and clinical examinations should be performed more frequently in these mild UNE patients.

There are many clinical classifications and questionnaires related to UNE **[7-9]** but few neurophysiological classifications in the literature **(10,11)**. There is an article on neurophysiological classification using nerve conduction study and needle EMG findings **(11)**. In this article, this classification was described in two cases. In our study, abnormal needle EMG findings were present in more than half of mild UNE patients and all moderate and severe UNE patients. In addition, as we have just mentioned, needle EMG abnormalities can be seen due to axonal damage or motor conduction block. Therefore, a neurophysiological classification involving needle EMG findings may not be useful. However, needle EMG is useful in showing axonal degeneration in some mild UNE patients or in differential diagnosis. UNE patients with needle EMG abnormalities should be closely monitored.

There were some limitations in our study. First, the symptom duration of the patients was variable. In patients with a symptom duration of 1 month, MUPs with chronic neurogenic changes may develop later. Second, the number of mild UNE patients with abnormal and normal needle EMG findings was low. However, it should be noted that patients with diabetes mellitus, carpal tunnel syndrome, or polyneuropathy were excluded from the study.

We think that it is impractical to use needle EMG findings in neurophysiological classification, as needle EMG abnormalities can be seen in most UNE patients and at each UNE stage. Abnormal needle EMG findings indicative of axonal damage or motor conduction block may be seen in mild UNE patients. Follow-up studies of these mild UNE patients will provide information about the prognosis of these patients. 

## Acknowledgments

This research did not receive any specific grant from funding agencies in the public, commercial, or nonprofit sectors.
